# Structural and Immunological Characterization of Novel Recombinant MOMP-Based Chlamydial Antigens

**DOI:** 10.3390/vaccines6010002

**Published:** 2017-12-25

**Authors:** Guillermo Madico, Olga Gursky, Jeff Fairman, Paola Massari

**Affiliations:** 1 National Emerging Infectious Diseases Laboratories, Boston University, Boston, MA 02118, USA; gmadico@bu.edu; 2 Department of Physiology & Biophysics and the Amyloidosis Treatment and Research Center, Boston University School of Medicine, Boston, MA 02118, USA; gursky@bu.edu; 3 SutroVax, Inc., Foster City, CA 94404, USA; jfairman@sutrovax.com; 4 Department of Immunology, Tufts University School of Medicine, Boston, MA 02111, USA

**Keywords:** vaccine, *Chlamydia*, MOMP, *neisseria*, PorB, porins, TLR2

## Abstract

*Chlamydia* is the most common cause of bacterial sexually transmitted infections worldwide. While infections resolve with antibiotic treatment, this is often neglected in women due to frequent asymptomatic infections, leading to disease progression and severe sequelae (pelvic inflammatory disease, ectopic pregnancy, infertility). Development of a vaccine against *Chlamydia* is crucial. Whole organism-based vaccines have short-lived activity, serovar/subgroup-specific immunity and can cause adverse reactions in vaccinated subjects. The *Chlamydia* major outer membrane protein (MOMP) is a prime candidate for a subunit vaccine. MOMP contains four regions of sequence variability (variable domains, VDs) with B-cell and T-cell epitopes that elicit protective immunity. However, barriers for developing a MOMP-based vaccine include solubility, yield and refolding. We have engineered novel recombinant antigens in which the VDs are expressed into a carrier protein structurally similar to MOMP and suitable for recombinant expression at a high yield in a correctly folded and detergent-free form. Using a carrier such as the PorB porin from the human commensal organism *N. lactamica*, we show that PorB/VD chimeric proteins are immunogenic, antigenic and cross-reactive with MOMP. VDs are unique for each serovar but if combined in a single vaccine, a broad coverage against the major *Chlamydia* serovars can be ensured.

## 1. Introduction

*Chlamydia trachomatis* (*Ct*) is an obligate intracellular gram-negative organism that is the leading cause of bacterial sexually-transmitted infections and preventable blindness (trachoma) worldwide [1,2,3,4]. There are 15 major serovars of *Ct*: A, B, Ba and C are associated with trachoma; D–K are sexually-transmitted and cause genital, respiratory, gastrointestinal and ocular infections, and L1–L3 cause lymphogranuloma venereum [5,6]. The most common acute clinical manifestations of *Chlamydia* infections include urethritis, epididymitis in males and cervicitis in women. Antibiotic therapy is available, but it does not prevent recurring infections and there is a concern that it may hinder the development of natural immunity [7,8]. In addition, since women are asymptomatic in most cases, the need for treatment is not always recognized, leading to progression into severe persistent infections with long-term sequelae such as pelvic inflammatory disease (PID), ectopic pregnancy and infertility [9,10]. *Ct* infection also increases HIV transmission and human papilloma virus–induced neoplasia [11,12]. The best approach to control this pathogen is by vaccination; however, development of a clinically protective vaccine for *Chlamydia* remains a global challenge [13,14,15,16,17]. 

Production of specific, neutralizing antibodies is the major goal of vaccines against bacterial infections [18] but, since *Chlamydia* is an obligate intracellular pathogen, strong and long-lasting T-cell responses, particularly CD4^+^ T-cell mediated, are also necessary for pathogen clearance [19,20,21]. Whole organism-based vaccines, both live and inactivated, have been tested for protection against *Ct*-induced trachoma, but they only elicited short-lived and serovar/subgroup-specific protective immunity and, in some vaccinated individuals, hypersensitivity reactions following exposure to *Ct* have also been reported [22,23,24]. Alternative strategies based on subunit vaccines have been explored using mainly *Chlamydia* outer membrane components [13]. 

The major outer membrane protein (MOMP) is a prime vaccine candidate. MOMP, which constitutes about 60% of the *Chlamydia* outer membrane protein content, is a surface-exposed trimeric porin with a putative 16-stranded β-barrel transmembrane core region, 8 surface-exposed loops and 8 short periplasmic loops per monomer [25,26,27,28]. Molecular characterization and a topology modeling of MOMP have identified four serovar-specific domains of sequence variability (variable domains, VD) in loops 2, 3, 5 and 6, along with constant domains (CDs) [26,29]. The VDs contain B- and T-cell epitopes which can elicit humoral responses (monoclonal and polyclonal) and the CDs can induce T-cell responses [30,31,32,33,34,35]. A large majority of studies of MOMP as a vaccine antigen have been carried out using MOMP from *Chlamydia muridarum* (*Cm*) (previously called *C. trachomatis* mouse pneumonitis) [36]. The clinical course of *Cm* genital infection in mice closely parallels that of *Ct* in humans, providing a clinical context for immunization/challenge studies [37,38,39]. *Cm* MOMP has been tested as a vaccine antigen in animal models of genital or respiratory *Chlamydia* challenge [40,41,42,43,44].

Unfortunately, development of a native MOMP-based vaccine has been delayed by significant challenges in production and scale-up of this protein. First, purification of this intrinsic membrane protein requires detergents, which is a major barrier for use in humans, due to toxicity. A second major bottleneck for scaling up production of nMOMP is due to the intracellular nature of *Chlamydia*, a limiting factor for growing large amounts of this organism. Production of recombinant MOMP has been attempted but, due to its hydrophobic and cysteine-rich nature, correct refolding has not been achieved, with subsequent loss of conformational epitopes. Vaccines based on unfolded rMOMP or a number of other alternatives (i.e., peptides and synthetic multi-epitope antigens [45,46,47], DNA plasmids, delivery vectors [48,49,50], nanoparticles and vesicles [51,52,53], and even expression in transgenic carrots and rice [54,55]) have only been partly successful [44,56,57]. 

A simplified rMOMP production process is needed to improve the expression efficiency, the yield and quality of the protein and, importantly, refolding without the loss of protective conformational epitopes. We have designed novel chimeric antigens by expressing MOMP VDs into a recombinant carrier protein that is structurally similar to MOMP and is suitable for high yield, refolding and scale-up production. Entire MOMP loops containing VD1, VD2, VD3 or VD4 were placed into the surface-exposed loops of the outer membrane protein PorB from the commensal organism *Neisseria lactamica* (NL) and were expressed recombinantly. Here, we present evidence that the PorB/VD proteins fulfill structural requirements for induction of immune responses to MOMP and are suitable for inclusion in a vaccine against *Chlamydia*. 

## 2. Materials and Methods 

### 2.1. Bioinformatics Analyses

The DNA and protein sequences of PorB from *N. lactamica* (NL) strain Y92-1009 [58] and of *C. muridarum* (*Cm*) MOMP [32] were used as reference to design the PorB/VD constructs. The protein sequences were analyzed with bioinformatics tool ClustalW2 [59] for identification of suitable residues for insertion/swapping of entire loops of MOMP containing VD1, VD2, VD3 or VD4 into the loops of NL PorB.

### 2.2. Cloning Strategy

Two PCR products were generated containing *porB* DNA sequences upstream and downstream of the designated loops to be swapped, and a third PCR product containing the VD region of MOMP to be inserted, with primers containing *porB* sequences in their 5′ ends. The three oligomers were joined by overlapping PCR to generate products containing the desired NL PorB DNA sequence containing the MOMP VDs using our previously described methodology [60,61]. The resulting PCR products were cloned into a TA PCR vector and sequenced (Tufts DNA Core Facility, Boston, MA, USA) before cloning into an expression vector for protein expression in *E. coli* BL21 cells. Expression of PorB/VD chimeric proteins clones were examined by gel electrophoresis. PorB/VD4 (MOMP loop 6 containing VD4 inserted into NL PorB loop 4) and PorB/VD2 (MOMP loop 3 containing VD2 inserted into NL PorB loop 6) were generated in this way, while subsequent cloning of PorB/VD1 (MOMP loop 2 containing VD1 into NL PorB loop 7), PorB/VD3 (MOMP loop 5 containing VD3 into NL PorB loop 5) and of NL PorB was performed by gene synthesis and sub-cloning (Genscript, Piscataway, NJ, USA).

### 2.3. Recombinant Expression and Purification of PorB/VDs

pET17b plasmids encoding for recombinant PorB/VDs or NL PorB were used for transforming *E. coli* BL21 (DE3) as previously described [62]. Clones were screened by antibiotic selection on LB agar plates with carbenicillin (50 µg/mL) at 37 °C in a 5% CO_2_ atmosphere, and individual colonies were picked for subsequent culture overnight in liquid LB with carbenicillin (100 µg/mL). To confirm the presence of *porB*/*VD* genes, culture aliquots (5 mL) were used for DNA extraction (Miniprep kits, Qiagen, Germantown, MD, USA), digested with NdeI and BamHI and examined on a 2% agarose gel. To confirm protein expression, culture aliquots (5 mL) were induced with IPTG (0.2 mM final concentration) for 1 h at 37 °C, lysed and examined by SDS-PAGE. Positive cultures were stored at −80 °C in glycerol stocks. For protein expression, cultures were scaled up to 300 mL and incubated overnight to a final OD_600_ 0.6–1.0 prior to induction with IPTG for 3–4 h. Protein extraction from inclusion bodies and chromatography purification was carried out as previously described [58]. Briefly, bacterial pellets were suspended in TEN (50 mM Tris HCl, 1 mM EDTA, 100 mM NaCl pH 8.0) buffer, lysed with lysozyme and deoxycholate in the presence of PMSF, followed by DNase I treatment and sonication to remove bacterial DNA. After centrifugation, pellets were sonicated in TEN with 8M urea followed by addition of Zwittergent 3–14 prior to column chromatography. *E. coli* LPS content was assessed by SDS-PAGE and Silver staining, and quantified by LAL assay (ThermoScientific) as EU/mL per the manufacturer’s specification. Purification of native and recombinant *Cm* MOMP was carried out as previously described [27].

### 2.4. Molecular Modeling

Predicted structural models of PorB/VD and NL PorB monomers and trimers were created with SWISS MODEL Workspace [63,64] and NGL Viewer [65]. As a template, the 3.2 Å resolution *X*-ray crystal structure of the *N. meningitidis* PorB was used (PDB ID 3WI4, 63% amino acid sequence homology with NL PorB) [66]. 

### 2.5. Structural Analyses 

#### 2.5.1. Electrophoresis

Purified PorB/VDs and NL PorB were examined by conventional SDS-PAGE and by modified SDS-PAGE in non-denaturing conditions as previously described [67]. Equal amounts of protein were dissolved in sample buffer with or without SDS and were either incubated for 5–10 min at 100 °C or kept at 25 °C. Bands corresponding to different molecular-weight forms corresponding to monomers, dimers, trimers and oligomers were visualized by Coomassie staining. 

#### 2.5.2. Circular Dichroism Spectroscopy

To evaluate the protein secondary structure and thermal stability, circular dichroism (CD) data were recorded using an AVIV 450 spectropolarimeter (AVIV BioMedical, Lakewood, NJ, USA) with Pelletier temperature control. Far-UV CD spectra were recorded at 185–250 nm from solutions of 0.16–0.2 mg/mL protein concentration in 10 mM NaPO_4_ (pH 8.0) containing 0.05% Zwittergent 3–14. The samples were placed in 1 mm path length cells; spectra were recorded with a 1 nm step size with an accumulation time of 30 s/nm and averaged over two consecutive scans. First, a spectrum from each sample was recorded at 25 °C. Next, thermal stability was assessed by sample heating from 25 °C to 98 °C at a constant rate of ~70 °C/h followed by consecutive cooling; heat-induced secondary structural unfolding was monitored at 197 nm. Thereafter, the second consecutive spectrum was recorded from the same sample at 25 °C. This was followed by protein incubation at 95 °C for 5 min and recording of the third consecutive spectrum at 95 °C. These experiments were recorded in technical triplicates to ensure reproducibility. Data analysis and display was performed using ORIGIN software (MicroCal, Northhampton, MA, USA).

### 2.6. Cell Cultures and Stimulations

The HEK293 NF-κB/SEAP-reporter cells (HEK-Blue) (InvivoGen, San Diego, CA, USA) expressing human TLR2/CD14 (HEK-Blue-hTLR2) or TLR4/MD-2/CD14 (HEK-Blue-hTRL4) were cultured in complete DMEM (10% heat-inactivated FBS, glutamine, 50 U/mL penicillin/50 μg/mL streptomycin and 100 μg/mL Normocin) plus 1× HEK-Blue selection medium at 37 °C in a 5% CO_2_ atmosphere. For stimulation, 10^5^ cells/mL were seeded in 96-well plates in 100 µL of HEK-Blue Detection medium and incubated for 14–16 h with medium alone purified PorB/VDs and NL PorB (10 μg/mL), Pam_3_CSK_4_ (100 ng/mL), ultra-pure *E. coli* LPS (100 ng/mL) and recombinant human TNF-α (50 ng/mL) (all from InvivoGen). Stimulations were performed in triplicate wells and each experiment was repeated at least three times. Expression of secreted embryonic alkaline phosphatase (SEAP) expression was detected spectrophotometrically at OD_620_ directly in the supernatants of stimulated cell cultures. 

### 2.7. Vaccine Preparation and Immunization of Mice 

Female C57BL/6 mice (4–6 weeks old, Jackson Laboratory, Bar Harbor, ME, USA) were housed and cared for in accordance with National Institutes of Health (NIH) and Tufts University IACUC protocols. Purified NL PorB and each PorB/VD construct were used to immunize mice combined with the adjuvants CpG DNA 1826 (InvivoGen) and Montanide ISA 720 (Seppic, Paris, France), in a final volume of 100 μL/mouse. Antigens (10 μg/mouse) were first mixed with CpG DNA (10 μg/mouse) and then with Montanide ISA 720 at a ratio of 30:70 (*v*/*v*) to obtain a homogeneous water-in-oil emulsion [68]. As a mock-vaccination control, PBS (100 μL/mouse) was also used. Mice (PorB/VD4, *n* = 5; all other groups *n* = 8) were immunized subcutaneously three times at two-week intervals. Pre-immune sera were collected by sub-mandibular bleed prior to immunization and immune sera two weeks after each immunization (week 2, week 4 and week 6). Sera were stored at −80 °C until use.

### 2.8. Antibody ELISA 

Antigen-specific serum antibody levels were measured by ELISA using plates coated with 2 μg/mL of PorB/VDs, NL PorB, rMOMP and nMOMP as previously described [69]. Serial dilution of pre-immune and immune sera from individual mice immunized with PorB/VDs and NL PorB were tested in duplicate wells, and pooled sera from mice immunized with rMOMP [41] were tested in triplicate wells. Alkaline-phosphatase (AP) conjugated secondary anti-mouse total IgG and IgG subclasses antibodies (Sigma, St. Louis, MO, USA) and 1 step PNPP substrate (Pierce) at O.D.405 were used for detection as specified by the manufacturer. A reference standard curve was used to quantify the amount of total IgG and IgG subclasses in µg/mL using a linear regression function. The individual Ig subclasses ratios were calculated by dividing the IgG2b, IgG2c or IgG3 by the IgG1 and the Th1:Th2 index was calculated as ((IgG2b + IgG2c + IgG3)/3)/IgG1. The ELISA for antibody cross-reactivity with *Chlamydia* elementary bodies (EBs) was performed in the laboratory of Prof. L.M.de la Maza (University of Irvine, CA, USA) as previously described [68]. Briefly, sera were tested in plates coated with 1 µg/well of EBs from UV-killed *C. muridarum* (strain Nigg II; ATCC VR 123) and results were expressed as geometric mean titers (GMT) determined using as a background the OD of preimmunization sera ± 2SD.

### 2.9. Cytokine ELISA

INF-γ and IL-12p70 (Th1 cytokines), IL-4 and IL10 (Th2 cytokines), IL-6 and TNF-α in the mouse sera were examined by ELISA using Opt-EIA kit (BD Biosciences, San Jose, CA, USA) specific for each cytokine according to the manufacturer’s protocol. Cytokines were measured in sera from individual mice from each group tested in duplicate wells, expressed in pg/mL and normalized to the cytokine amounts measured in pooled pre-immune sera from each group. The Th1:Th2 index was calculated as INF-γ/IL-4.

### 2.10. Statistical Analyses

The analyses were carried out with GraphPad Prism using parametric and non-parametric tests (unpaired *t*-test and Mann Whitney test, indicated by *) for comparisons of two samples/conditions, and one-way or two-way analyses of variance (ANOVA) with Tukey’s multiple comparisons test (indicated by ^≈^) to determine significance between groups. Differences were considered significant at a minimum *p* value of <0.05, as indicated in the text and in the figure legends.

## 3. Results 

### 3.1. MOMP and PorB Sequence Analyses

**Figure 1:**
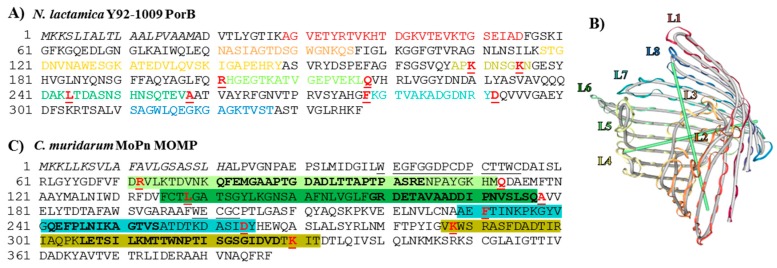
Cloning Strategy. (**A**) Sequence of *N. lactamica* Y92-1009 PorB. The leader sequence is indicated in italic. Loops are indicated as follows: **L1**: Dark red. **L2**: Orange. **L3**: Ochre. **L4**: Yellow. **L5**: Lime green. **L6**: Green. **L7**: Teal. **L8**: Blue. Residues in L4, L5, L6 and L7 for insertion of MOMP VDs are in bold underlined red; (**B**) Cartoon of the NL PorB monomer (side view) created with Swiss-Model server [63,64] and NGL Viewer 0.9.0 [65] based on the *Nme* PorB template (PDB ID 3WI4) [66]. The loops are color-coded as in (**A**). (**C**) Sequence of *C. muridarum* MOMP. The leader sequence is in italic. Loops are indicated as follows: **L1**: Underlined. **L2**: Lime green box with residues corresponding to VD1 bolded. **L3**: Green box, VD2 residues bolded. **L4**: Underlined. **L5**: Teal box, VD3 residues bolded. **L6**: Yellow box, VD4 residues bolded. Residues in L2, L3, L5 and L6 for insertion into PorB loops are in bold underlined red.

The amino acid sequences of NL PorB (Figure 1A) and *Cm* MOMP (Figure 1C) were examined to identify common residues in the surface-exposed loops of these proteins for genetic manipulation. Based on structural and function features of PorB, loops 2 and 3 were excluded from mutagenesis due to their direct role in the pore-forming function (Figure 1A,B, L2: Orange, L3: Ochre) [70,71]. Evidence from the *N. meningitidis* PorB structure has suggested that L1 (Figure 1A,B, L1: Dark red) may be involved in inter-trimer contacts [66], and this loop was also assigned a low priority for mutagenesis. In contrast, loops 4, 5, 6, 7 and 8, which are not located at the trimeric interface, are less likely to destabilize the protein structure. Ultimately, L4, L5, L6 and L7 were chosen as recipients for MOMP VDs, based on identification of residues in the loop-flanking regions that were suitable for VDs insertion (Figure 1A; bold, underlined red). The amino acid sequence of MOMP, the loops and the VDs are shown in Figure 1C. The PorB/VD proteins were constructed as follows: NL PorB L5 was swapped with MOMP L2 containing VD1 (PorB/VD1); MOMP L3 containing VD2 was inserted into NL PorB L6 (PorB/VD2); NL PorB L7 was swapped with MOMP L5 containing VD3 (PorB/VD3) and MOMP L6 containing VD4 was inserted in NL PorB L4 (PorB/VD4).

### 3.2. Molecular Representation

**Figure 2:**
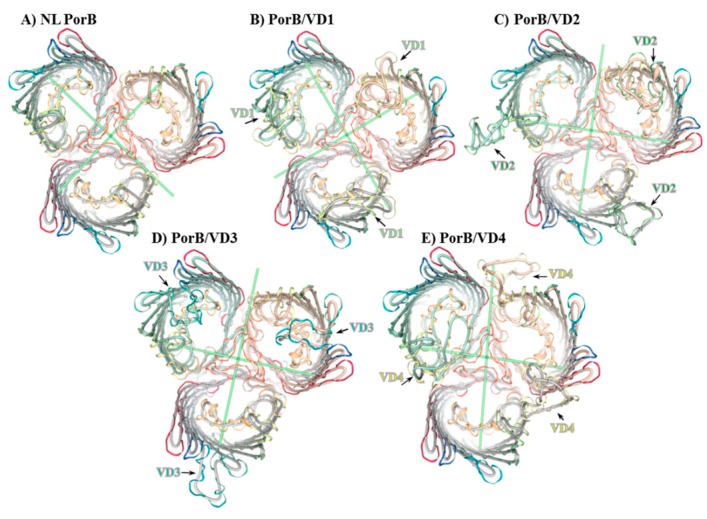
Molecular representation of PorB/VDs. Cartoon of predicted trimers (top view) created with Swiss-Model server [63,64] and NGL Viewer 0.9.0 [65] based on the *Nme* PorB template (PDB ID 3WI4) [66]. Loops are depicted as follows: L1: Red. L2: Orange. L3: Ochre. L4: Yellow. L5: Lime green. L6: Green. L7: Teal. L8: Blue. (**A**) NL PorB; (**B**) PorB/VD1: NL PorB L5 (lime green) is replaced with MOMP L2 containing VD1; (**C**) PorB/VD2: NL PorB L6 (green) is replaced with MOMP L3 containing VD2; (**D**) PorB/VD3: NL PorB L7 (teal) is replaced with MOMP L5 containing VD3; (**E**) PorB/VD4: NL PorB L4 (yellow) is replaced with MOMP L6 containing VD4.

In the absence of X-ray crystallographic structures, NL PorB and PorB/VDs were modeled on the *N. meningitidis* PorB template (PDB ID 3WI4) previously published by our group [66], using Swiss-Model server and NGL Viewer [63,65]. A cartoon of the NL PorB monomer is shown in Figure 1B, and trimers are shown in Figure 2A–E. While the PorB/VD models reflect the best prediction criteria for the spatial positioning of each VD into NL PorB, variability of loop structures and orientation of each mutant can only be verified by future high-resolution structural analyses, such as X-ray crystallography or cryo-EM. 

### 3.3. Cloning and Expression of Recombinant PorB/VDs 

**Figure 3:**
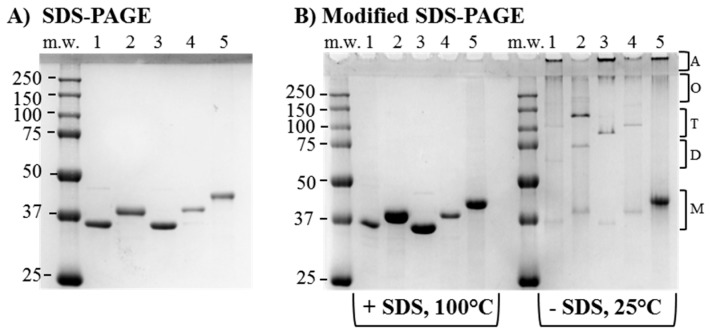
Electrophoretic analysis of purified PorB/VDs. Coomassie staining of (**A**) Conventional SDS-PAGE. Lane 1: NL PorB (~34 kDa); Lane 2: PorB/VD1 (~37 kDa); Lane 3: PorB/VD2 (~35 kDa); Lane 4: PorB/VD3 (~37 kDa); Lane 5: PorB/VD4 (~38 kDa); (**B**) Modified SDS-PAGE. Left panel: samples dissolved in loading buffer with SDS and incubated at 100 °C for 5 min; Right panel: samples dissolved in SDS-free loading buffer and kept at 25 °C prior to electrophoresis Lane numbers are marked as in (**A**). The predicted positions of bands of molecular weight corresponding to monomers (M), dimers (D), trimers (T), oligomeric forms (O) and aggregates (A) are indicated by the brackets.

The recombinant proteins were expressed in *E. coli* strain BL21 (DE3) into inclusion bodies and chromatographically purified free of *E. coli* LPS contamination as previously described [58,67]. Throughout the purification, the porin-containing fractions were monitored by conventional SDS-PAGE and Coomassie staining, and ultimately pooled for inclusion into endotoxin-free, detergent-free proteosome micelles. Bands of molecular weight corresponding to each predicted monomeric form were observed by SDS-PAGE (Figure 3A), with a shift in the size of the NL PorB monomer (Figure 3A, lane 1) from approximately 34 kDa to about 37 kDa for PorB/VD1, 35 kDa for PorB/VD2, 37 kDa PorB/VD3 and 38 kDa for PorB/VD4 (Figure 3A, lanes 2, 3, 4 and 5), consistent with loops insertion/replacement. No significant *E. coli* LPS content was detected by SDS-PAGE and silver staining, and LAL assay showed LPS content under 3.0 EU/mL for all samples.

### 3.4. Structural Analyses

Using a modified electrophoresis in the absence of SDS in the gel, our group and others have previously shown that a reduction in the amount of PorB monomers is accompanied by the presence of bands corresponding to dimers, trimers and complexes of high molecular weight [67]. Aliquots of equivalent protein concentration (3.5–5 µg/lane) of NL PorB, PorB/VD1, PorB/VD2, PorB/VD3 and PorB/VD4 were dissolved in either SDS-free loading buffer and kept at 25 °C (to prevent both detergent and thermal denaturation), or in SDS-containing loading buffer and heated at 100 °C for 5 min (for complete protein denaturation). In SDS-free electrophoresis, the fully-denatured samples (+SDS, 100 °C) on a SDS-free gel revealed bands of molecular weight corresponding to the monomeric forms of each protein (Figure 3B, left panel). In contrast, non-denatured samples (−SDS, 25 °C) showed a significantly reduced monomer population and a corresponding increase of higher molecular-weight populations consistent with dimers (68–76 kDa, depending on the protein), trimers (102–114 kDa), and higher molecular-weight oligomers (>250 kDa) including large aggregates trapped in the stacking gel wells (Figure 3B, right panel). PorB/VD4 showed a larger monomer population in non-denaturing conditions than the other proteins (Figure 3B, right panel, lane 5), suggesting that the insertion of MOMP L6-VD4 into L4 of NL PorB may destabilize the trimer. 

**Figure 4:**
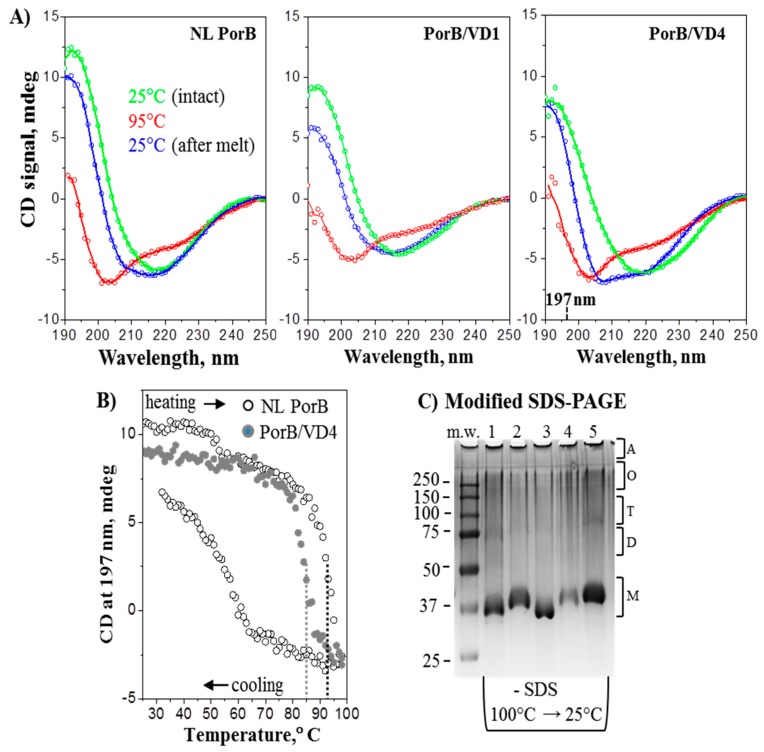
Secondary structure and thermal stability of PorB/VDs. (**A**) Far-UV CD spectra of NL PorB, PorB/VD1 and PorB/VD4 (left to right) representative of the PorB/VDs. In each panel, three consecutive spectra were recorded of the same sample (0.18 mg/mL protein in 10 mM sodium phosphate and 0.05% Zwittergent 3–14 at pH 8.0). Green lines: intact sample at 25 °C. Blue lines: sample was heated to 98 °C and cooled to 25 °C, followed by data collection at 25 °C. Red lines: sample was incubated at 95 °C for 5 min and the data was recorded at 95 °C. (**B**) Representative thermal denaturation data recorded at 197 nm during sample heating from 25 °C to 98 °C, followed by cooling to 25 °C at a rate of 70 °C/h. Sample conditions are as in panel A. The directions of the temperature changes are indicated. Vertical dotted lines indicate the midpoints of thermal unfolding. (**C**) Coomassie staining of modified SDS-PAGE. Samples were dissolved in SDS-free loading buffer, heated at 100 °C for 5 min. and cooled to 25 °C prior to electrophoresis. Lane 1: NL PorB; Lane 2: PorB/VD1 Lane 3: PorB/VD2; Lane 4: PorB/VD3; Lane 5: PorB/VD4. The predicted position of bands of molecular weight corresponding to monomers (M), dimers (D), trimers (T), oligomeric forms (O) and aggregates (A) are indicated.

To characterize the secondary structure and thermal stability of the PorB/VDs, CD spectroscopy was used. Far-UV CD spectra of samples in 0.05% Zwittergent 3–14 recorded at 25 °C revealed that all proteins had the expected predominantly β-sheet structure, evidenced by a negative CD peak centered circa 217 nm and a positive CD peak circa 193 nm, consistent with the PorB β-barrel structure. Scans of NL PorB and PorB/VD1 and PorB/VD4 are shown in Figure 4A (green lines) and are representative of PorB/VD2 and PorB/VD3 as well. Slight spectral variations observed between NL PorB and the PorB/VDs were likely due to presence of the VDs. Next, protein thermal stability was assessed upon heating to 98 °C and consecutive cooling to 25 °C at a constant rate, as described in the Methods. CD spectra of each protein at 95 °C showed a complete secondary structural unfolding, with a negative peak circa 203 nm characteristic of a random coil (Figure 4A, red lines). The spectra recorded at 25 °C after heating to 98 °C showed partial refolding of the secondary structure (Figure 4A, blue lines). However, refolding was incomplete, as evidenced by the discrepancy between the spectra recorded from the same sample before and after heating (see Figure 4A, green and blue lines). 

The CD melting data were consistent with this observation and showed a discrepancy between the heating and cooling curves; such a hysteresis is a hallmark of thermodynamically irreversible unfolding. Representative heating and cooling data of NL PorB recorded by CD at 197 nm illustrate this effect (Figure 4B, open symbols). Similar results were obtained for the PorB/VDs. Comparison of the heating data revealed that PorB/VD4 (Figure 4B, closed symbols) unfolded at significantly lower temperatures that the other PorB/VDs or NL PorB (Figure 4B, dotted lines). Thus, the apparent midpoint of the thermal unfolding for PorB/VD4 was observed at 86 ± 1.5 °C, while it was at 94 ± 1.5 °C for NL PorB, and 92–94 °C for PorB/VD1, PorB/VD2 or PorB/VD3. These results suggest that the loop exchange between NL PorB and MOMP and insertion of VD1, VD2 or VD3 in NL PorB had little effect on the protein stability, while the insertion of VD4 into PorB L4 was significantly destabilizing. Although temperature-dependent changes in far-UV CD spectra of PorB have been previously described by us and others [67,72], the current results provide the first evidence for the thermodynamic irreversibility of these structural changes. 

To further explore this irreversible thermal unfolding, the electrophoretic behavior of NL PorB and the PorB/VDs upon heating/cooling was examined by modified SDS-PAGE. Figure 4C shows a substantial heat-induced increase in the monomer population accompanied by a decrease in the population of oligomeric species, which was not reverted by cooling. This result is consistent with the thermodynamically irreversible thermal unfolding observed by CD. Together, these results reveal that recombinant NL PorB and PorB/VDs undergo thermal denaturation in a thermodynamically irreversible manner with an almost complete loss of the quaternary structure and partial loss of the secondary structure observed upon heating and cooling.

### 3.5. PorB/VDs Induce TLR2 Signaling and Are Free of LPS

**Figure 5:**
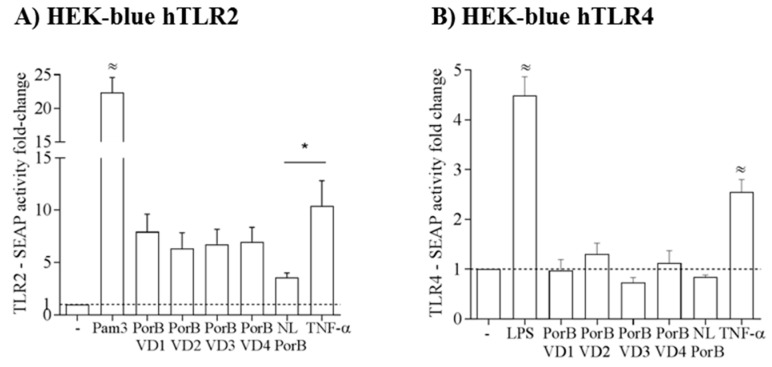
PorB/VDs induce TLR2 activation and are free of LPS. (**A**) HEK-blue-hTLR2 cells were incubated with medium alone, purified PorB/VDs and NL PorB (10 μg/mL), Pam_3_CSK_4_ (100 ng/mL) and TNF-α (50 ng/mL). ^≈^ *p* <0.0001 by one-way ANOVA with Tukey’s multiple comparisons test; (**B**) HEK-blue hTLR4 cell were incubated with NL PorB and PorB/VDs as in (**A**), and with *E. coli* LPS (100 ng/mL). SEAP activity was measured spectrophotometrically at OD_620_ directly in the plate after 14–16 h and is reported as fold-change relative to medium alone ± SEM. ^≈^ *p* < 0.0001 as above; * *p* < 0.05 by unpaired *t* test.

PorB is an established TLR2/TLR1 agonist, and when the trimeric structure of this protein is disrupted, its TLR2-dependent activity is significantly attenuated [58,67]. Previously, our group has also shown that NL PorB is a weaker inducer of TLR2-dependent IL-8 production by epithelial cells than the PorB from *N. meningitidis* and Pam_3_CSK_4_, via NF-κB only (for NL PorB) or NF-κB and AP-1 (for Nme PorB and Pam_3_CSK_4_) signaling, respectively. Here, a HEK-Blue-hTLR2 cell SEAP reporter system was used to assess the TLR2-dependent activity of NL PorB and PorB/VDs. Cells (10^5^ cells/mL) were incubated with medium alone, Pam_3_CSK_4_ (100 ng/mL) and NL PorB or PorB/VDs (10 μg/mL) for 14–16 h, SEAP activity was measured spectrophotometrically at OD_620_ and reported as fold-change relative to medium alone. The highest levels of SEAP were induced by Pam_3_CSK_4_ (^≈^ *p* significant by one-way ANOVA with Tukey’s multiple comparisons test) (Figure 5A). Consistent with our previous results, NL PorB-induced SEAP was low, but a trend of increased SEAP activity was observed for the PorB/VD constructs (Figure 5A). This suggests that the VDs insertion into NL PorB may enhance the molecular mechanisms of TLR2 signaling by this protein. Notably, although a higher monomer population had been observed for PorB/VD4 (see Figure 3B), it induced comparable TLR2-dependent SEAP activity than the other constructs. It is possible that PorB/VD4 monomer/trimer forms are in a different equilibrium state in modified electrophoresis and cell stimulation experiments. In HEK-Blue-hTLR4 cells, *E. coli* LPS (100 ng/mL) induced high SEAP production, while neither NL PorB nor PorB/VDs activated cells significantly above baseline levels (medium alone) (Figure 5B). Collectively, these results indicate low to negligible LPS content in the purified proteins, in agreement with the LAL assay. In both cell lines, incubation with TNF-α (50 ng/mL), a non-TLR dependent cell activation control, also induced SEAP activity (Figure 5A,B), and this was significantly higher than that induced by NL PorB in HEK-Blue-hTLR2 cells (* *p* significant by unpaired *t* test); TNF-α also induces IL-8 via both NF-κB and AP-1 [58] and possibly SEAP in HEK-Blue-hTLR2 cells via the same mechanisms.

### 3.6. Immunogenicity of PorB/VDs

**Figure 6:**
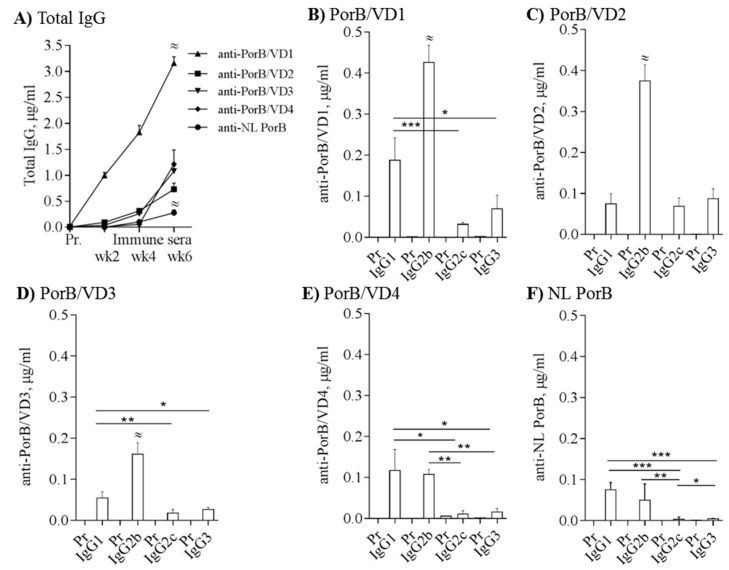
PorB/VDs are immunogenic. (**A**) Total serum IgGs measured by ELISA in sera from mice immunized with NL PorB or PorB/VDs plus CpG DNA and Montanide ISA 720 as adjuvants. PorB/VD1: upward triangles; PorB/VD2: squares; PorB/VD3: downward triangles; PorB/VD4: diamonds; NL PorB: circles. Pre-immune (Pr) and immune sera (week 2, week 4 and week 6) from individual mice in each group were tested in duplicate wells and are expressed as μg/mL ± SEM. ^≈^ *p* < 0.0001 by two-way ANOVA with Tukey’s multiple comparisons test; (**B**–**F**) IgG subclasses (μg/mL ± SEM) measured as above. ^≈^ *p* < 0.0003 by one-way ANOVA with Tukey’s multiple comparisons test. *; **; *** *p* < 0.05 by Mann-Whitney test.

C57BL/6 mice were immunized with 10 µg/mouse of NL PorB, PorB/VD1, PorB/VD2, PorB/VD3 (*n* = 8) or PorB/VD4 (*n* = 5) using CpG DNA 1826 (10 µg/mouse) and Montanide ISA 720 as adjuvants. These adjuvants have been previously used with nMOMP and rMOMP for induction of robust, protective humoral and cellular (CD4+ T cells and Th1-biased cytokines) immune responses [20,38,41,68,73]. Additional mice were immunized with PBS alone, as experimental controls for each set of PorB/VDs immunizations. Serum IgG responses were examined in the pre-immune sera (Pr) and in sera collected after each immunization (week 2, week 4 and week 6) by ELISA, and the antibody levels were quantified by extrapolation based on a mouse IgG standard curve [69]. Immunization with PorB/VD1 induced the highest levels of total IgGs, while NL PorB showed the overall lowest immunogenicity (Figure 6A, upward triangle and circle, respectively) (^≈^ *p* significant by two-way ANOVA with Tukey’s multiple comparisons test). PorB/VD2, PorB/VD3 and PorB/VD4 induced antigen-specific IgG responses of variable amplitude (Figure 6A square, downward triangle and diamond). No antigen-specific IgGs were detected in the pre-immune sera from mice in each immunization group (Figure 6A) or in sera from PBS-immunized mice.

Next, IgG subclasses were measured. PorB/VD1, PorB/VD2 and PorB/VD3 induced significantly high IgG2b levels (Figure 6B,D) (^≈^ *p* significant by one-way ANOVA with Tukey’s multiple comparison test) and variable amounts of IgG1, IgG2c and IgG3 (Figure 6B,D) (*^,^**^,^*** *p* significant by Mann-Whitney test). In contrast, PorB/VD4 and NL PorB induced comparable levels of IgG1 and IgG2b (Figure 6E,F) and lower levels of IgG2c and IgG3. IgG1 production is generally considered to correlate with Th2 responses, while IgG2s and IgG3 are considered Th1-dependent antibodies. Based on the IgG subclasses results, the ratio of IgG2b/IgG1, IgG2c/IgG1 and IgG3/IgG1 was calculated and a Th1:Th2 index was extrapolated as an indication of Th bias (Table 1). For example, based on the IgG ratios for PorB/VD2 and PorB/VD3, the resulting Th1:Th2 index >1 would suggest a potential stronger Th1-biased antibody response than to PorB/VD4 and NL PorB, for which the Th1:Th2 index was <1, even though the same adjuvants were used for immunizations. 

### 3.7. Serum Cytokines 

**Figure 7:**
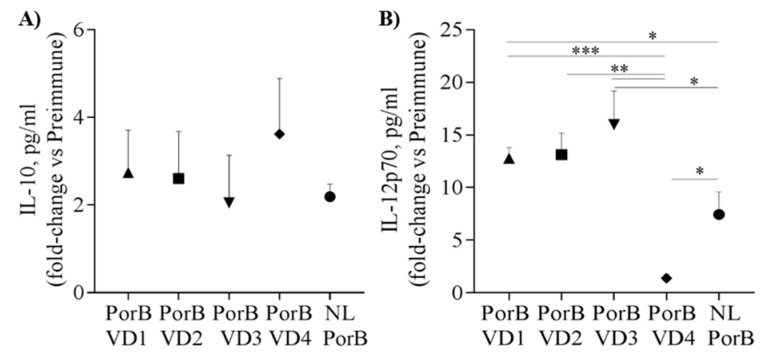
Immunization with NL PorB and PorB/VDs induces different cytokine responses. Serum cytokine levels were measured by ELISA and quantified in pg/mL. Values from immune sera of individual mice (week 6) in each group were normalized to the pooled pre-immune sera from the same group and expressed as fold-change ± SEM. (**A**) IL-10 and (**B**) IL-12p70. *; **; *** *p* < 0.05 by unpaired *t*-test.

The cytokine pattern in sera from immunized mice was also examined by ELISA. Cytokine levels (pg/mL) in immune sera from individual mice in each group were normalized to the pooled pre-immune sera from each group and reported as pg/mL fold-change. NL PorB and PorB/VDs induced overall similar levels of IL-10 (Figure 7A); in contrast, PorB/VD1, PorB/VD2 and PorB/VD3 induced significantly more IL-12p70 than PorB/VD4 and NL PorB (Figure 7B) (*; **; *** *p* significant by unpaired *t* test). Next, IL-4 and IFN-γ were examined. NL PorB and the PorB/VDs induced comparable levels of IL-4 (Table 2), while PorB/VD1, PorB/VD2 and PorB/VD3 induced more IFN-γ than PorB/VD4 and NL PorB. Cytokines are also considered a predictive surrogate of Th-type polarization. Based on the IFN-γ/IL-4 ratio, a Th1:Th2 index was calculated as a further indication of Th-bias (Table 2). Similar to the IgG subclasses results, a Th1:Th2 index >1 was obtained for PorB/VD1, PorB/VD2 and PorB/VD3, again suggesting a potential stronger Th1-type polarization than for PorB/VD4 and NL PorB (Th1:Th2 index < 1). However, T cell stimulation experiments will be necessary to complement and confirm these correlations. Lastly, the acute phase cytokines IL-6 and TNF-α were also examined. All antigens induced IL-6, particularly PorB/VD2 and PorB/VD4 (Table 2); TNF-α levels showed a greater variability, with considerably higher levels induced by PorB/VD1, PorB/VD3 and PorB/VD4. 

### 3.8. Cross-Reactivity and Cross-Immunogenicity

**Figure 8:**
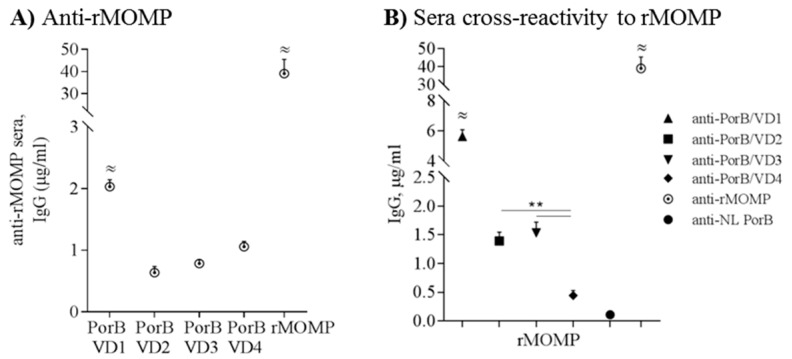
Antibodies to PorB/VDs and rMOMP are cross-reactive. (**A**) Anti-rMOMP (pooled mouse sera) tested against PorB/VDs and NL PorB by ELISA. IgG levels were measured in triplicate wells from two independent experiments and are expressed as μg/mL ± SEM. ^≈^ *p* significant by one-way ANOVA with Tukey’s multiple comparisons test; (**B**) Sera tested against rMOMP as above. Anti-PorB/VD1: upward triangle; anti-PorB/VD2: square; anti-PorB/VD3: downward triangle; anti-PorB/VD4: diamond; anti-NL PorB: circle; anti-rMOMP: dotted circle (positive control). Sera from individual mice in each immunization group were tested in duplicate wells. ^≈^ *p* significant as above; ** *p* < 0.005 by Mann Whitney test.

Having established that the PorB/VDs are antigenic, the specificity of the antibody responses was further parsed. First, anti-rMOMP mouse serum (a pooled sera aliquot) [41] was tested against each PorB/VD by ELISA to assess specific recognition of the VD components. Significantly higher anti-rMOMP IgG levels were measured against PorB/VD1 than against PorB/VD2, PorB/VD3 or PorB/VD4 (Figure 8A) (^≈^ *P* significant by one-way ANOVA with Tukey’s multiple comparisons test). As expected, high IgG levels were measured to rMOMP (Figure 8A) (^≈^ *p* significant as above), due to the sera being raised against this protein which contains all the VDs and other potential epitopes. Cross-reactivity was also measured against rMOMP using anti-PorB/VDs sera. The sera raised to PorB/VD1 recognized rMOMP significantly better than the sera to other PorB/VDs (Figure 8B, upward triangle) (^≈^ *p* significant as above); anti-PorB/VD2 and anti-PorB/VD3 sera recognized rMOMP similarly (Figure 8B, square, downward triangle) and significantly better than anti-PorB/VD4 (Figure 8B, diamond) (** *p* significant by Mann Whitney test). Anti-rMOMP sera recognized rMOMP efficiently (^≈^ *p* significant), while anti-NL PorB showed negligible cross-reactivity with rMOMP (Figure 8B, closed circle and dotted circle). Collectively, these results suggest that VD1 positioning into NL PorB L5 either favors an overall better immune exposure/recognition compared to other constructs, or that VD1 may be more immunogenic than the other VDs in the context of these antigens.

To assess whether the anti-PorB/VD sera specifically recognized MOMP in the bacterial membrane context, the sera were tested against *Cm* elementary bodies (EBs) and titers were compared to those against rMOMP. The highest titers against EBs and rMOMP were measured with anti-PorB/VD1 sera (Table 3); anti-PorB/VD3 titers against EBs were lower than to rMOMP, while anti-PorB/VD2 and anti-PorB/VD4 sera were low-titered against both antigens. It is possible that the low titers to the EBs are due to a different conformation of MOMP in the context of the bacterial membrane, or to different epitopes exposure/accessibility than in purified rMOMP. Consistent with the previous ELISA results using purified rMOMP, anti-NL PorB sera showed the lowest titers against both EBs and rMOMP (Table 3). 

**Figure A1:**
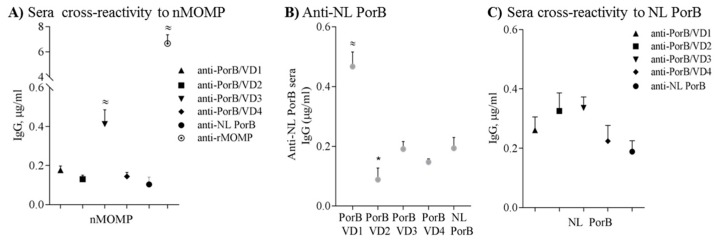
Antibodies to PorB/VD and nMOMP are cross-reactive and have limited cross-reactivity with NL PorB. (**A**) Anti-PorB/VD sera tested against nMOMP by ELISA. Sera from individual mice in each immunization group were tested in duplicate wells and IgG are expressed in μg/mL ± SEM. Anti-PorB/VD1: upward triangle; anti-PorB/VD2: square; anti-PorB/VD3: downward triangle; anti-PorB/VD4: diamond; anti-NL PorB: circle; anti-rMOMP: dotted circle. ^≈^ *p* < 0.0001 by one-way ANOVA with Tukey’s multiple comparisons test; (**B**) Anti-NL PorB sera tested against PorB/VDs and NL PorB as above. ^≈^ *p* < 0.0002 as above and * *p* < 0.05 by MannWhitney test; (**C**) Anti-PorB/VD sera tested against NL PorB as above.

Since MOMP in EBs is expected to be in native form, the anti-PorB/VDs sera were also tested against purified nMOMP. Recognition of nMOMP revealed a different profile than rMOMP, with overall lower IgG levels and better recognition by anti-PorB/VD3 instead than by anti-PorB/VD1 (Appendix Figure A1A) (^≈^ *p* significant by one-way ANOVA with Tukey’s multiple comparisons test). Recognition of nMOMP by anti-PorB/VD1 and anti-PorB/VD4 was only slightly higher than anti-PorB/VD2 and anti-NL PorB. Anti-rMOMP sera recognized nMOMP (Appendix Figure A1A, dotted circle) (^≈^ *p* significant as above), although lower IgG levels were observed as compared to rMOMP (see Figure 8B); this could be due to lack of recognition of nMOMP conformational epitopes. In summary, sera from mice immunized with PorB/VDs recognize the VD epitopes in both rMOMP and nMOMP, although epitopes conformation may play a role in antibody specificity. 

Lastly, to determine whether antibodies were elicited to the NL PorB antigen component, cross-reactivity of anti-NL PorB sera with the PorB/VDs was examined. This revealed overall low IgG levels (Appendix Figure A1B), with slightly higher amounts of cross-reactive antibodies to PorB/VD1 (^≈^ *p* significant as above) and lower amounts of cross-reactive antibodies to PorB/VD2 than to the other PorB/VD constructs (* *p* significant by Mann Whitney test). Similarly, low cross-reactive antibody levels were measured against NL PorB in the anti-PorB/VD sera (Appendix Figure A1C). These results suggested that the NL PorB component of the PorB/VD antigens has a low immunogenicity and elicits limited production of specific antibodies. A statistically significant difference was observed for NL PorB recognition by anti-PorB/VD3 and anti-NL PorB (** *p* <0.005 by Mann Whitney test, but the total amount of specific antibodies to NL PorB was consistent with low cross-reactivity. 

## 4. Discussion

In a novel approach for a MOMP-based vaccine, we have expressed the immunogenic VD regions of MOMP into a carrier protein that is structurally similar to MOMP and is suitable for recombinant expression and folding. The PorB porin from the human commensal organism *Neisseria lactamica*, which shares critical structural features with MOMP, was used as carrier [32,58,66]. By sequence analysis of MOMP and PorB, we identified common residues in the surface-exposed loops of these proteins suitable for genetic manipulation. Our loop replacement strategy was aimed at (1) introducing minimal variability to the β-barrel core structure of NL PorB, to ensure a correct monomer folding for each mutant and (2) avoiding dramatic changes in loop surface charge, length and orientation, to minimize potential disruption of the quaternary structure.

Residues in the loop-flanking regions of NL PorB loops 4 through 7 were selected for mutagenesis. These loops were not located in the pore region or at the trimeric interface and hence, were not expected to interfere with the monomer folding, quaternary structure and surface exposure of the VDs. By expressing individual VDs into each momomer of NL PorB, the resulting PorB/VDs express three copies of each VD. Models of the PorB/VDs, based on the available crystal structure of *N. meningitidis* PorB [66], suggested that the VDs positioning into the NL PorB loops was likely to result in immune-accessibility. The recombinant proteins were not toxic in *E. coli*, were easily expressed and purified by column chromatorgaphy with our methods optimized for PorB, and resulted in proteins of molecular weight consistent with the expected size of NL PorB plus the corresponding VDs. Loop net charge analysis of each protein showed minor changes; specifically, the net charge of NL PorB L5, which was +1 [+4/−3], shifted to +2 [+7/−5] for NL PorB/VD1; L6 shifted from −1 [+1/−2] to −2 [+2/−4] in PorB/VD2; L7 shifted from +1 [+3/−2] to 0 [+4/−4] in PorB/VD3, while L4 remained +1 ([+2/−1] to [+6/−5]). However, future co-expression of multiple VDs or VD combinations may introduce more significant loop surface charge variations, which will need to be considered for construction of VD mosaic antigens aiming at expanding the breadth of vaccine strains coverage. Biochemical and biophysical evaluation of the secondary and quaternary structure of the PorB/VDs was generally consistent with the prototypical PorB structure, such as the β-sheet and trimer formation. However, PorB/VD4 revealed a higher population of monomers under non-denaturing conditions. CD spectroscopic data also showed a decreased thermal stability for PorB/VD4 as compared to other proteins explored. These results suggest that the replacement of NL PorB L4 with MOMP L6 containing VD4 introduces unexpected steric hindrance at the monomer-monomer interface of the trimer, thereby destabilizing it. In the future, alternative cloning strategies can be explored based on the numerous common residues in MOMP and PorB, or using other PorB variants with variable-length loops for added flexibility. Further X-ray crystallographic or electron microscopy studies will define the structure of PorB/VDs with the best antigen potential. Some variability in the proteins thermostability was observed as compared to previous analyses of PorB from other *Neisseriae* [72]. The current CD spectra and electrophoretic analyses showed that NL PorB and the PorB/VDs undergo a thermodynamically irreversible thermal denaturation; a more detailed analysis of the kinetic barriers that contribute to porin stability is needed to address these findings. 

PorB is a TLR2 ligand whose activity depends on a correctly folded quaternary structure [58,66,67,74]. Hence, the induction of TLR2 signaling provided an indirect evidence of the desired structural features of the PorB/VDs. Introduction of the VDs into NL PorB loops increased TLR2-dependent SEAP activity in a HEK cell reporter model as compared to NL PorB. Considering that MOMP also has a weak TLR2-dependent activity *in vitro* [75], it is possible that VDs insertion into NL PorB loops may enhance the protein’s critical interactions with TLR2, resulting in amplification of signaling. However, the molecular mechanisms of TLR2-dependent signaling by MOMP are not known and the role of the VDs remains speculative. In addition, the nature of each PorB/VD antigen could have a different effect for the molecular mechanisms of immunity linked to TLR2 signaling; for example, although all PorB/VDs are antigenic, variability in the humoral and systemic response was observed. Specifically, IgG responses to PorB/VD1 were greater than those to other PorB/VDs and to NL PorB, indicating different immunogenic consequences for each construct. Variability in the quantity and the type of IgG subclass composition was observed: IgG2b dominated the response to PorB/VD1, PorB/VD2 and PorB/VD3, together with an IgG3 component; in contrast, PorB/VD4 and NL PorB induced similar levels of IgG1 and IgG2b were induced by, with low IgG2c and IgG3. Since IgG subclasses are influenced by and influence cytokine production [76], the serum cytokine pattern was also examined and revealed a similar propension for an apparent Th1 biased response for PorB/VD1, PorB/VD2 and PorB/VD3, with elevated levels of IL-12p70 and IFN-γ, while all the constructs induced similar systemic Th2 cytokines (IL-4 and IL-10). It is unclear whether these antigens may have an intrinsic Th bias, or if PorB/VD4 and NL PorB may drive a stronger Th2 response that leads to Th1 suppression (particularly via IL-10), and thus a potential apparent Th2 bias. However, only analysis of the T cell responses, in particular CD4+ cells, following immunization with PorB/VDs will reveal whether a specific Th bias defines each PorB/VD antigen. Ultimately, antigens that are capable of inducing CD4+ T cells with Th1-biased cytokines, mainly IFN-γ, along with humoral responses, can induce optimal responses for protection against *Chlamydia* infections and would be highly desirable [35].

Immune responses to vaccination are not only influenced by adjuvants, but also by intrinsic antigen structural features, or modulotypes [77]. The finding that PorB/VD1, PorB/VD2 and PorB/VD3 appear to favor Th1-biased responses despite being used with the same adjuvants as PorB/VD4 and NL PorB, could be due to a contribution of NL PorB itself to the vaccine. As a TLR2 ligand, NL PorB has immune adjuvant properties and favors Th1-Th2 mixed responses with a stronger Th2 component [69]. Thus, the effect of NL PorB combined with that of Montanide ISA 720 may explain the resulting Th2 propension for this antigen. However, when VD1, VD2 and VD3 are introduced into NL PorB, some NL PorB modulotypes may be altered, resulting in unanticipated Th1-Th2 biasing immune events during immunization. This effect may not happen for VD4 introduced into NL PorB, with responses more consistent with those of NL PorB. Future studies with different adjuvant types and/or doses may improve and direct the response to the desired humoral and Th-type cellular immunity. Notably, specific recognition of the VDs by sera from immunized mice was higher than that of the NL PorB component of each antigen. The VDs were also recognized by sera raised in mice against rMOMP, suggesting that the linear epitopes were presented for immune recognition. Interestingly, anti-rMOMP cross-reactive antibodies to PorB/VD1 were higher than those to other PorB/VDs, suggesting that either VD1 was more immunogenic, or that its positioning into L5 of NL PorB favored immune exposure. In that case, it is possible that placement of other VDs in the same NL PorB loop would lead to similar results. Importantly, antibodies to PorB/VD1 specifically recognized MOMP in the context of *Chlamydia* elementary bodies (EBs), the infectious form of this organism. However, cross-reactivity of anti-PorB/VD sera with a purified native *Cm* MOMP was unexpectedly lower than to the EBs (or rMOMP). Using nMOMP, anti-PorB/VD1 sera were surpassed by anti-PorB/VD3 sera in the response amplitude; this finding suggests that the epitope conformation will need to be further evaluated. Analysis of the protective potential of PorB/VDs in the mouse model of immunization and *C. muridarum* challenge is currently ongoing.

**Figure A2:**
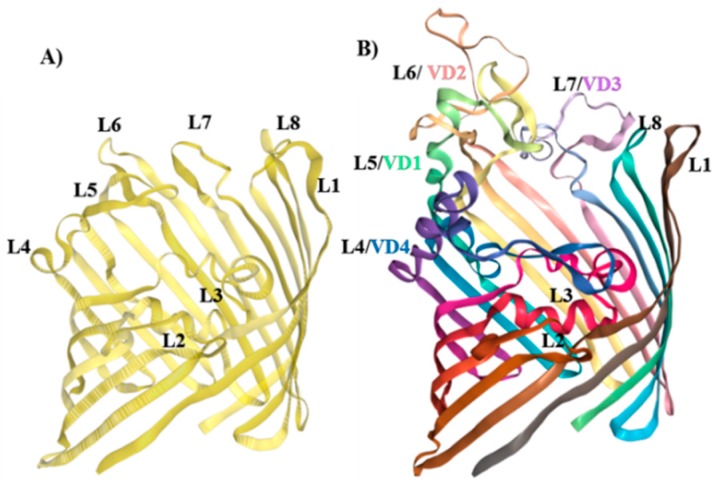
Molecular representation of NL PorB/multiple VDs. **(A**) Cartoon of NL PorB monomer (side view) created with Swiss-Model server [63,64] and NGL Viewer 0.9.0 [65] based on the *Nme* PorB template (PDB ID 3WI4) [66]. Loops 1 through 8 are indicated; (**B**) Cartoon of the predicted PorB/VD1-VD2-VD3-VD4 monomer. PorB L1: dark brown; L2: light brown; L3: cherry; L4 replaced with MOMP L6 containing VD4: purple/blue; L5 replaced with MOMP L2 containing VD1: green/yellow; L6 replaced with MOMP L3 containing VD2: peach/pink; L7 replaced with MOMP L5 containing VD3: lavender; PorB L8: teal.

It is possible that expressing multiple VDs into NL PorB will lead to higher specificity and a comprehensive coverage of all the epitopes, particularly the conformational ones. The trimeric nature of PorB combined with interchangeability of the VDs will allow a variety of combinations. For example, super-antigens expressing multiple copies of a single VD (such as the constant region of VD4, TTLNPTIAG), individual VDs of MOMPs from the major human *Ct* serovars, or even all the VDs simultaneously could be desirable for broad coverage against multiple strains. A cartoon of a NL PorB construct in which L4, L5, L6 and L7 are replaced with Cm MOMP VD4, VD1, VD2 and VD3 is shown in Appendix Figure A2, Supplementary Materials. In addition, incorporation of VDs from other *Chlamydia* species, for example *C. pecorum*, a major pathogen for the koala (*Phascolarctos cinereus*, an endangered species), will also improve veterinary vaccine strategies against infection [78]. 

Induction of antibodies to the NL PorB component in mice may not be relevant, since they are not naturally colonized by *Neisseriae*. However, it may be a concern in humans, since *N. lactamica* is a common human nasopharyngeal commensal carried by a large majority of individuals, especially early in life [79,80]. It is thought that this organism may also contribute to natural immunity against *N. meningitidis,* but whether immunity to *N. lactamica* has a negative impact on commensal carriage and/or natural immunity is still not well defined [81,82,83]. Our results in mice show low antibody levels to NL PorB and suggest that our loop replacement strategy enhanced immune responses to VDs but not to NL PorB. Moreover, by carefully selecting the carrier PorB, it may be possible to develop a multifunctional antigen by inserting the VDs into the PorB from another major sexually-transmitted pathogen, *Neisseria gonorrhoeae*. In conclusion, these initial studies support the feasibility of our approach for obtaining MOMP-based novel recombinant antigens that are suitable for scale-up production in detergent-free, folded, immunoreactive and immunogenic form. 

## 5. Conclusions

Numerous strategies are being explored to develop a vaccine against *Chlamydia* infections, but no definitive formulation has been identified so far. Despite MOMP is one of the most promising protective antigens for a subunit vaccine, hurdles concerning its production in a structurally stable form suitable for scale-up are still unresolved. Our approach is based on design of recombinant antigens composed of the immunogenic VD regions of MOMP expressed into the Neisserial PorB porin as a carrier. We show that the chimeric PorB/VD antigens are suitable for scale-up production in detergent-free, folded, immunoreactive and immunogenic form. Antibodies raised to the PorB/VDs are cross-reactive with MOMP, in particular to the VD1 and VD3 regions. Immunization with PorB/VD1 and PorB/VD3 combined with CpG DNA/Montanise as adjuvants appears to favor IgG subclasses and cytokines associated with Th1-biased responses, while PorB/VD4 showed a higher propension for Th2-biased IgG subclasses and cytokines. Studies in the mouse model of *Chlamydia* genital challenge will reveal if such responses are protective against infection. A major advantage of our strategy is the opportunity to engineer superantigens composed of MOMP VD regions from multiple *Chlamydia* serovars for broad coverage against the major *Chlamydia* serovars simultaneously.

## Figures and Tables

**Table 1:** IgG subclasses ratio (µg/mL ± SEM).

Ratio	PorB/VD1	PorB/VD2	PorB/VD3	PorB/VD4	NL PorB
IgG2b/IgG1 ^a^	3.5 ± 0.85	8 ± 1.6	4.1 ± 1.1	2 ± 0.69	2.48 ± 2.23
IgG2c/IgG1 ^a^	0.27 ± 0.06	1.42 ± 0.41	0.46 ± 0.15	0.11 ± 0.02	0.41 ± 0.4
IgG3/IgG1 ^a^	0.32 ± 0.08	2.38 ± 0.81	0.73 ± 0.19	0.41 ± 0.26	0.14 ± 0.05
Th1:Th2 index ^b^	0.935	2.348	1.254	0.388	0.194

**^a^** IgG subclasses ratio measured as IgG2b/IgG1, IgG2c/IgG1 and IgG3/IgG1 in µg/mL. ^b^ Th1:Th2 index measured as (IgG2b + IgG2c + IgG3 (µg/mL)/3)/IgG1 (µg/mL).

**Table 2:** Immunization with NL PorB and PorB/VDs induces different cytokine responses.

Cytokine	PorB/VD1	PorB/VD2	PorB/VD3	PorB/VD4	NL PorB
IL-4 ^a^	1.19 ± 0.55	3.56 ± 0.7	3.1 ± 0.72	2.29 ± 0.09	2.57 ± 0.67
IFN-γ ^a^	5.44 ± 0.2	10.05 ± 4.79	3.65 ± 0.25	2.08 ± 0.62	2.84 ± 1.09
Th1:Th2 index ^^^	2.85	2.81	1.80	0.90	1.10
IL-6 ^a^	5.23 ±0.82	16.63 ± 3.47	7.6 ± 1.12	11.14 ± 2.56	4.81 ± 1.7
TNF-α ^a^	7.87 ± 1.78	1.58 ± 0.67	5.9 ± 0.68	12.75 ± 3.63	0.46 ± 0.24

**^a^** Cytokines fold-change measured as immune sera/pre-immune sera (pg/mL ± SEM). ^ Th1:Th2 index measured as IFN-γ (pg/mL)/IL-4 (pg/mL).

**Table 3:** ELISA antibody titers to *Cm* UV-inactivated EBs or rMOMP using two-fold serial dilutions of pooled sera from immunized mice.

Sera	*Cm* EBs	rMOMP
Anti-PorB/VD1	12,800	>12,800
Anti-PorB/VD2	100	<100
Anti-PorB/VD3	200	3200
Anti-PorB/VD4	<100	100
Anti-NL PorB	<100	<100

## Supplementary Materials

Supplementary File 1
