# Supplementary material for: Structural and Immunological Characterization of Novel Recombinant MOMP-Based Chlamydial Antigens
Source: Vaccines (Basel). 2017 Dec 25;6(1):2. doi: 10.3390/vaccines6010002 (PMC5874643; doi:10.3390/vaccines6010002)
Supplement: Supplementary File 1 [file vaccines-06-00002-s001.zip › Madico et al. - supplemental figures 12 01 17.pptx]

## Slide 1
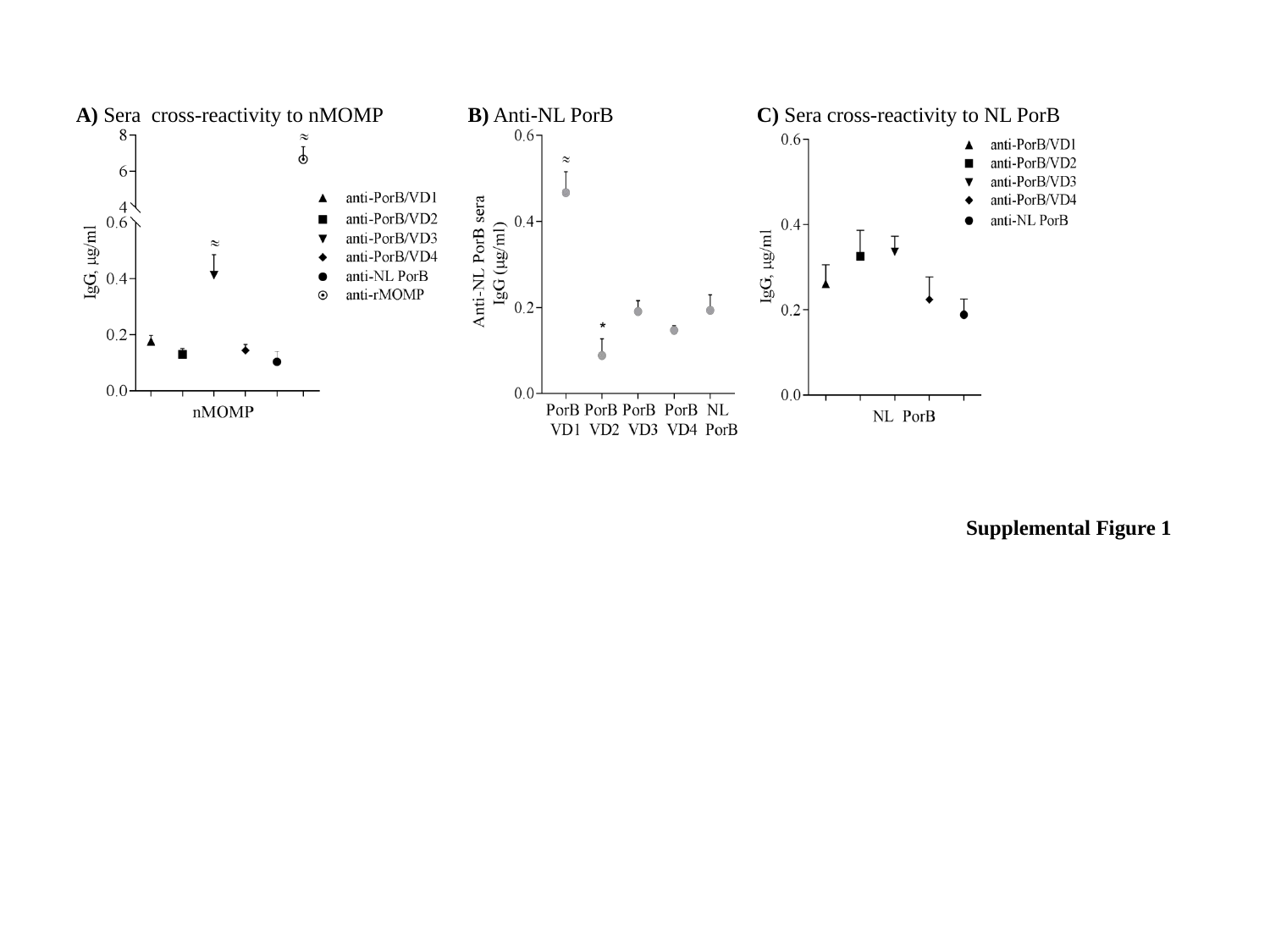

A) Sera cross-reactivity to nMOMP B) Anti-NL PorB C) Sera cross-reactivity to NL PorB
Supplemental Figure 1

## Slide 2
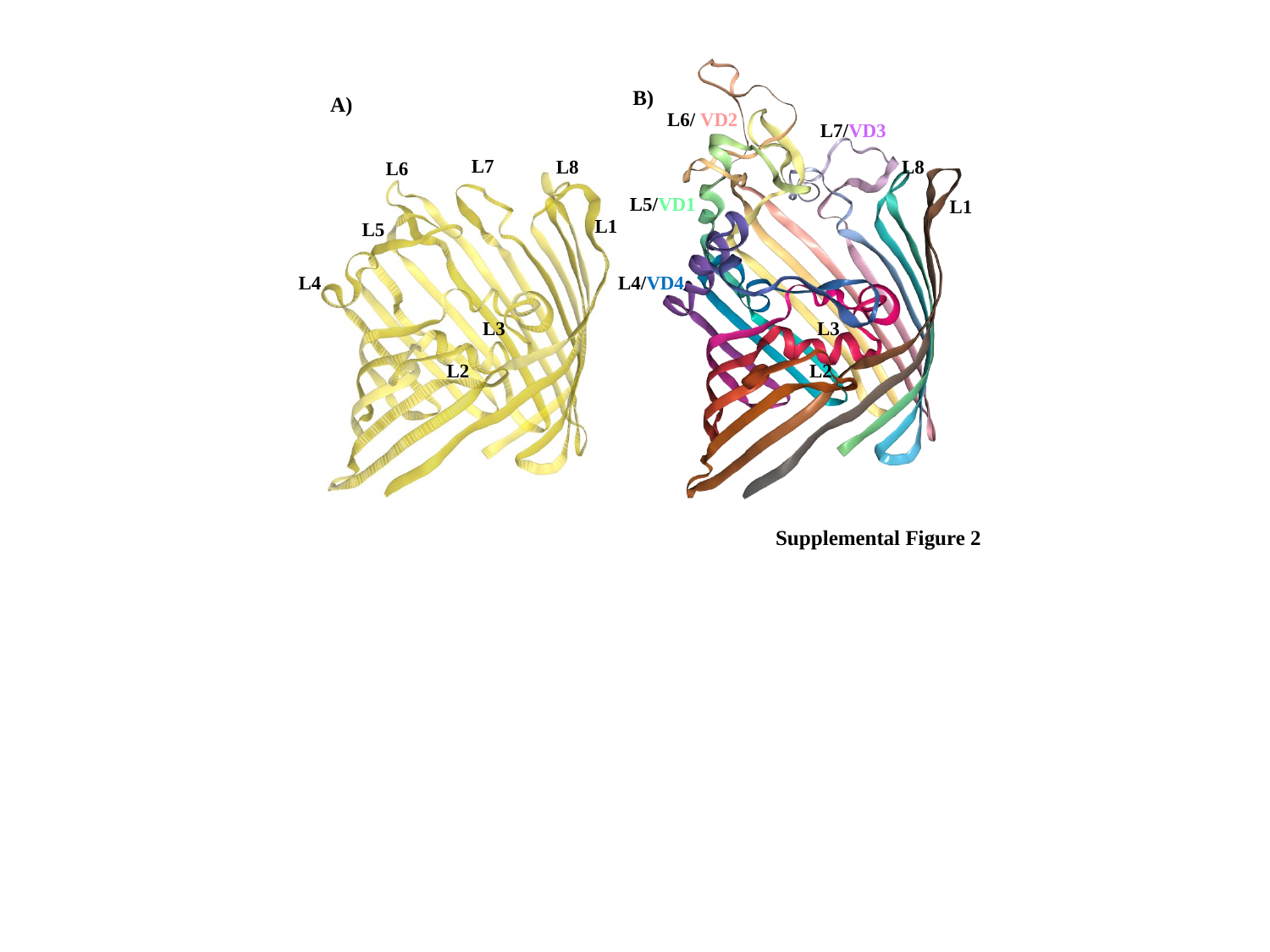

B)
A)
L6/ VD2
L7/VD3
L7
L8
L8
L6
L5/VD1
L1
L1
L5
L4
L4/VD4
L3
L3
L2
L2
Supplemental Figure 2
